# Recent Updates in Vaccine Delivery through Microneedles

**DOI:** 10.34172/apb.2023.001

**Published:** 2022-05-06

**Authors:** Kasturi Pawar

**Affiliations:** Exelixis Inc. Alameda, CA, USA 94502

**Keywords:** Vaccine delivery, Microneedles, Regulatory challenges, Formulation challenges, Stability

## Abstract

Recent coronavirus pandemic and its global socio-economic impact has re-emphasized the need for safe, fast, and efficient delivery of vaccines for humankind. With advent of technological advances, and to improve patient acquiescence, several techniques for fast, effective, and safe delivery of vaccines have been researched and published in the literature in last three decades. These delivery enhancement techniques include but are not limited to electroporation, microneedles (MN), ultrasound, iontophoresis, etc. This review aims at discussing the current research undergoing in vaccine delivery, specifically focusing on microneedles assisted, the historical background of microneedles and their introduction to drug delivery area, and a special focus on formulation challenges and stability in these systems. The review also sheds light on regulatory challenges one must keep in mind for bringing a successful microneedles-based vaccine delivery into market as well as a snapshot of current commercially available microneedles-based products in cosmetic and pharmaceutical industry.

## Introduction

 In drug delivery and pharmaceuticals, oral delivery is more widely accepted by patients and bio/pharmaceutical industry as compared to other routes of delivery owing to several benefits that it offers over others, especially injectables. These include a well-established delivery system, patient acquiescence, convenience, cost effectiveness, and noninvasiveness.^1,2^ Historically and even in current age, solid orals remain the most accepted dosage form for oral delivery, as also depicted by the percentage of industry involvement (percentage of bio/pharmaceutical companies developing oral dosage forms and academic research undergoing in oral delivery). Nevertheless, extensive research has been done in exploring novel systems such as micro/nano-particles, lipid-based systems, nose-to-brain delivery, etc.^3-7^ for drugs that are either difficult to deliver by oral routes or for better pharmacological performance. Specifically, if the indication is localized to skin, then other than the invasive hypodermic needle, microneedles (MNs, non-invasive) accessing target site as compared to topical therapies.

 Apart from the most acceptable route, delivery through skin has always been a fascinating route for researchers for its distinct benefits over other routes, such as non-invasiveness, elimination of first-pass effect, reduction of adverse effects, and greater patient compliance.^8^ Nevertheless, topical/transdermal delivery is not as easy since stratum corneum poses a main barrier for drug permeation. Vaccines, as compared to small molecule drugs that are synthetically derived are challenging in terms of delivery through skin owing to their large molecular weight, polarity, and risk of degradation by skin enzymes. Delivery of vaccine with skin pre-treatment opens avenues for a much higher and better delivery than topical or transdermal route without any assistance (passive delivery).^9-13^

 Various techniques have been used since years to improve transdermal drug delivery which include, the use of chemicals, iontophoresis, and MNs, to name a few.^14-17^ Combination of these techniques have also been reported with major success in improving the drug delivery across skin.^18-20^ This review article will focus on recent updates and strides in MN assisted drug delivery focusing on vaccine delivery.

## Current research and updates in microneedles mediated vaccine delivery

 A study reported by a group of researchers investigated an inactivated influenza vaccine (IIV) in a microneedle patch (MNP) for delivery of IIV in a Ph-I human trial, in terms of safety, reactogenicity, and immunogenicity. The clinical trial included 100 cohorts aged between 18 to 49 years and randomized to 4 groups of 25 per arm: (1) IIV by MNP administered by healthcare worker (HCW), (2) IIV by MNP self-administered by study participants, (3) IIV by IM injection administered by HCW or (4) placebo by MNP administered by HCW. Four questionnaires were administered: at Day 0 before and after study product delivery, and at Days 8 and 28. Compliance reported by subjects with MNP vaccination was 98.6% and 86.4% for IM vaccination. In terms of safety and compliance, MNP faired higher than the traditional vaccination.^21^

 Lanza and group have manufactured dissolvable MNs with LiHyp1- antigen along with TLR9 agonist, CpG, and compared with a cationic liposomal product containing the same antigen and agonist but delivered intravenously. The results indicated an elevated immune reaction and high levels of defense against L. donovani in MNs formulation in comparison to liposomal formulation. The study was performed in BALB/C mice and was characterized by measuring T cell responses, parasitic load in hepatic system and spleen, and antibody responses. These dissolvable MNs presented a great option for delivery, albeit a through stability/shelf life and scale up study needs to be evaluated in future.^22^

 Stinson and group investigated a MNP (MIMIX), made with silk fibroin for vaccine tips that implants itself in the skin, while releasing the influenza virus toxin for a few weeks, thus representing the usual timeline of a typical infection from this virus. The antigens that were utilized for this study include A/Michigan/45/2015 (H1N1), A/ Hong Kong/4801/2014 (H3N2), and B/Brisbane/60/2008 (B Victoria lineage). For 2018–19 vaccine, influenza antigens included A/ Michigan/45/2015 (H1N1), A/Singapore/INFIMH-16–0019/2016 (H3N2), and B/Maryland/15/2016 (B/Colorado/6/2017-like virus, B Victoria lineage). Silk fibroin solution was prepared from Bombyx mori silkworm fibers, and the MIMIX device, containing a 11x11 array of vaccine-loaded, insoluble silk fibroin tips was molded on dissolvable polymer casts. On administration, the casts disintegrate rapidly, thus leaving the silk tips inserted into the dermis for sustained delivery of vaccine payload over a period (14 days).^23^

 Chen et al utilized the MNs technology for delivering vaccines in animals for prevention of plague.^24^ F1 protein of Yersinia pestis (YP), which is the key moiety for eliciting protection was loaded into polyethylene glycol (PEG)-modified lipoparticles. The lipoparticles were inserted in immunocompromised Balb/c mice using MN. Cytokine level determination IL-4, IFN-γ and TNF-α along with IgG levels indicated that these tiny needles helped in an efficient delivery of vaccine. Further, challenging the mice with the toxic YP strain (Yokohama-R 00703) kept all mice protected indicating that the vaccine delivery was successful and efficient.

 Dissolving MNs fabricated from chitosan, poly(vinyl alcohol) and poly(vinyl pyrrolidone) were loaded with bovine serum albumin and proteolipid protein, PLP_139-151 _for peptide delivery through skin for treating multiple sclerosis. Drug loading for bovine serum albumin was high, and PLP_139-151 _co-localized with the chitosan in the MNs structure as indicated after labeling them with a fluorescent tag. The release of PLP peptide reported to be sustained with 40% released over 4 days, and within therapeutic doses thus promising a future potential drug delivery system for peptide delivery.^25^

 With the need of hour with COVID-19 pandemic, Kim and group investigated to deliver Middle East respiratory syndrome coronavirus (MERS-CoV) through dissolving CMC MNs.^26^ Adenovirus proteins, MERS-S1f, MERS-S1fRS09, MERS-S1ffliC, SARS-CoV-2-S1, or SARS-CoV-2-S1fRS09) were utilized. In-vivo study in BABL/c mice, after 6 weeks displayed that serum levels of immunized animals demonstrated not only a long-lasting but also substantial levels of virus neutralizing activity. This indicates a better antigen activity as compared to typical stratum corneum delivery, despite of the adjuvant. Additionally, the antigenicity of γ-exposed MNs vaccines was similar to unexposed MN thus suggesting stability and potential delivery system for MNs assisted SARS-CoV-2 subunit vaccines.

 To combine a bolus with a sustained release, Kim et al manufactured dissolvable MN with hepatitis-B vaccine (HBsAg). For the bolus dose, CMC formulation was coated on whereas for slow release it was plain polylactic acid (PLA) tips. These MNs achieved similar clinical efficacy as that of two separate shots of conventional IM administration.^27^ The bolus dose released within 20 min, when tested in vitro in female BALB/C mice. The PLA tips slowly dissolved and released the antigen by hydrolytic degradation over 55 days, thus providing an excellent combination of bolus and sustained delivery for a potential delivery system for vaccines.

 Jeon et al has developed a compartmental microneedle array (CMA) for attaining simultaneous delivery of two influenzas vaccines, however delivering at the same time as a single product, thus avoiding multiple injections. PLA MNs were coated with two strains of influenza vaccines, B/Yamagata (B-Y) and B/Victoria (B-V), in two compartments within the microneedles system, without physical mixing. Weight delta and survival made the key evaluating parameters in female BALB/C mice and CMA was compared with (a) combined vaccines with MN, (b) two monovalent vaccines with MN, (c) IM with a combined vaccine, and (d) IM with two monovalent vaccines. The CMA mice group demonstrated better survival and weight delta than other two groups. When challenged with Yamagata, the survival rate and body weight for all the MN groups were 100%, however, for control phosphate buffered saline and IM administered, B-Y + B-V were 0% and 60%, respectively. CMA group demonstrated relatively higher or equivalent survival rate and weight change as compared to IM administered B-Y + B-V. This opens avenue for a futuristic dual delivery system using these CMA techniques.^28^

 Delivery of influenza virus in VaxiPatch^TM^ containing subunit glycoprotein that act as vaccine antigens along with adjuvants in a MN system is reported by Ellison and group at Verndari Inc. rHA of influenza virus B/Colorado/06/2017 was combined with synthetic virosomes (SV). Further QS-21 from *Saponaria quillaja *was used to formulate adjuvant liposomes that either contained SV or not. This was further made with trehalose and dye for better stability and detection. Stainless steel MN arrays were utilized for this study. In-vivo study conducted in Sprague Dawley rats that were administered VaxiPatches^TM^ containing 0.3 mg of rHA, 0.5 mg QS-21 and 0.2 mg 3D - (6-acyl) PHAD and dye, demonstrated 100-fold higher toxoid-specific immunoglobulin-G titers as compared to 4.5 mg of FluBlok (*P* = 0.001) provided by IM route. Further, stability studies of these VaxiPatches^TM^ under accelerated conditions suggested retention of virosomal antigenic activity for at least two months at 60℃.^29^

## Conclusion

 MNs are not any novel technique to skin delivery, and span in areas of pharmaceutical and cosmeceuticals. Extensive research has been done in MNs-assisted vaccine delivery, alone or in combination with other techniques, and has produced encouraging results. Yet, from a commercial standpoint, not a lot of MNs-based products make to the market on annual basis. This is attributed to the formulation as well as scale up challenges that these systems pose, which makes it challenging from regulatory perspective as well. With the rise in several start-ups and funding available, along with clarity on regulations from US, EU, and other country-specific authorities, it is expected that in next 5 years, more MNs-based drug and vaccine delivery products/platform technologies will get approval, and commercial reality of this technique in par with conventional dosage form such as tablets and capsules, is not very far in future.

## Ethical Issues

 Not applicable.

## Conflict of Interest

 No conflict of Interest.
